# P-185. Seroprevalence and Clinical Spectrum of *Toxocara* Infections: A Retrospective Study from a Tertiary Care Center in Lebanon

**DOI:** 10.1093/ofid/ofae631.389

**Published:** 2025-01-29

**Authors:** L’Emir Wassim El Ayoubi, Nour El Meski, Samar Hassani, Nayda Bidikian, Jad Bou Khalil, Souha S Kanj

**Affiliations:** MedStar Health, Baltimore; American University of Beirut Medical Center, Beirut, Beyrouth, Lebanon; American University of Beirut Medical Center, Beirut, Beyrouth, Lebanon; Harvard Medical School, Beirut, Beyrouth, Lebanon; American University of Beirut Medical Center, Beirut, Beyrouth, Lebanon; American University of Beirut Medical Center, Beirut, Beyrouth, Lebanon

## Abstract

**Background:**

*Toxocara* is a prevalent zoonotic disease worldwide. It exhibits a broad spectrum of clinical manifestations, ranging from asymptomatic cases to severe conditions like myelitis and, in rare instances, fatal outcomes such as aortic thrombosis. Despite its prevalence, studies focusing on the seroprevalence, clinical manifestations, and laboratory findings of *Toxocara* in the MENA region are limited, highlighting a significant gap in research.

**Figure:**
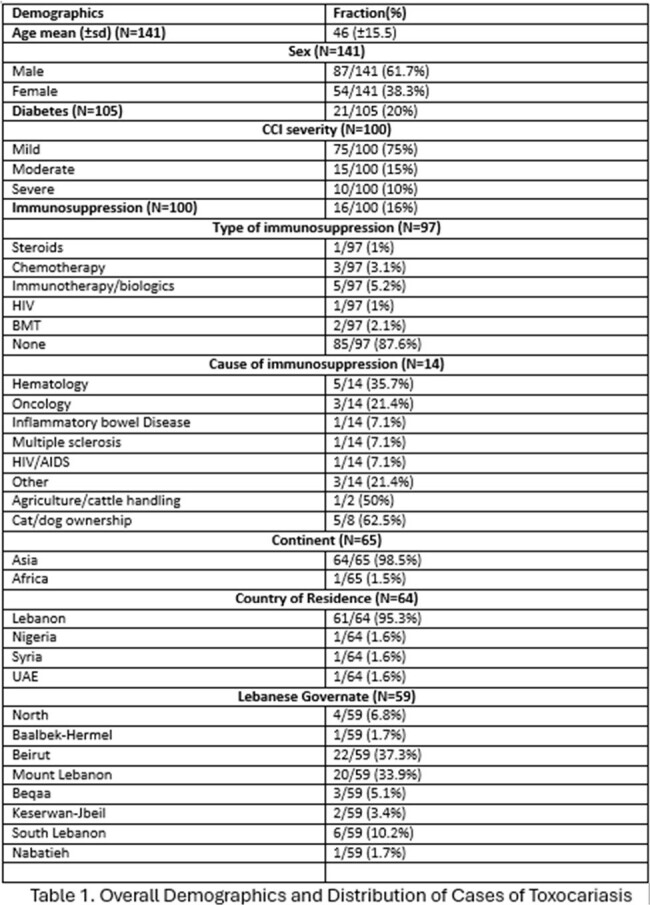

**Methods:**

This is a retrospective, single-center cohort study conducted at the American University of Beirut Medical Center. The charts of 227 patients who were tested for *Toxocara* via ELISA IgG, presenting either as inpatients or outpatients from January 2002 to September 2022, were examined.

**Figure:**
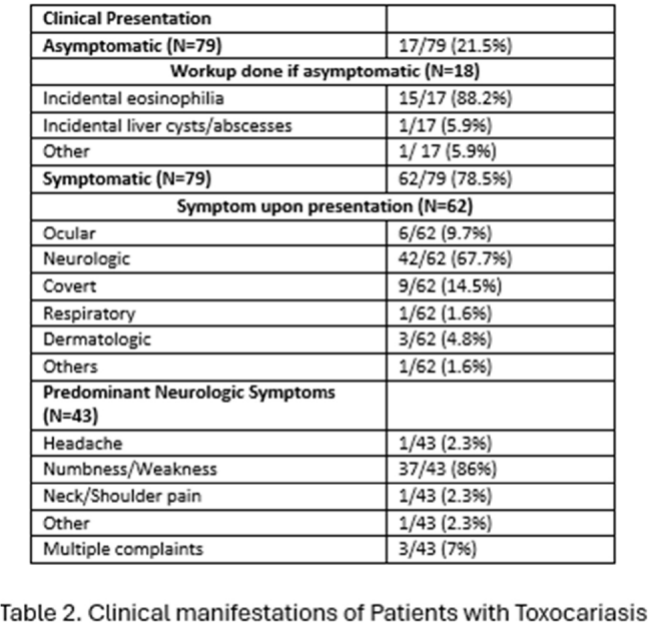

**Results:**

The majority of the patients were middle-aged, generally exhibiting few co-morbidities as evidenced by 75% (75/100) of the study population being classified as “mild” on the Charlson co-morbidity index and only 16% (16/100) being immunosuppressed. 95.3% (61/64) of the patients were residents of Lebanon, most of whom were living in either Beirut or Mount Lebanon (22/59 (37.3%) and 20/59 (33.9%), respectively). 78.5% (62/79) of the patients presented with symptoms, and 67.7% (42/62) primarily complained of neurologic symptoms, mostly tingling or weakness in the extremities (37/43 (86%)). Asymptomatic patients were typically investigated for incidental eosinophilia (15/17 (88.2%)). Except for a lower overall age (44 vs 55, p=0.005), no statistically significant variable was detected between symptomatic and asymptomatic patients in terms of demographic characteristics. Among patients with *Toxocara* myelitis, most of the MRIs examined revealed enhancing (7/8 (87.5%)) longitudinal lesions (6/10 (60%)), mainly involving the thoracic levels of the spine (4/10 (40%)).

**Figure:**
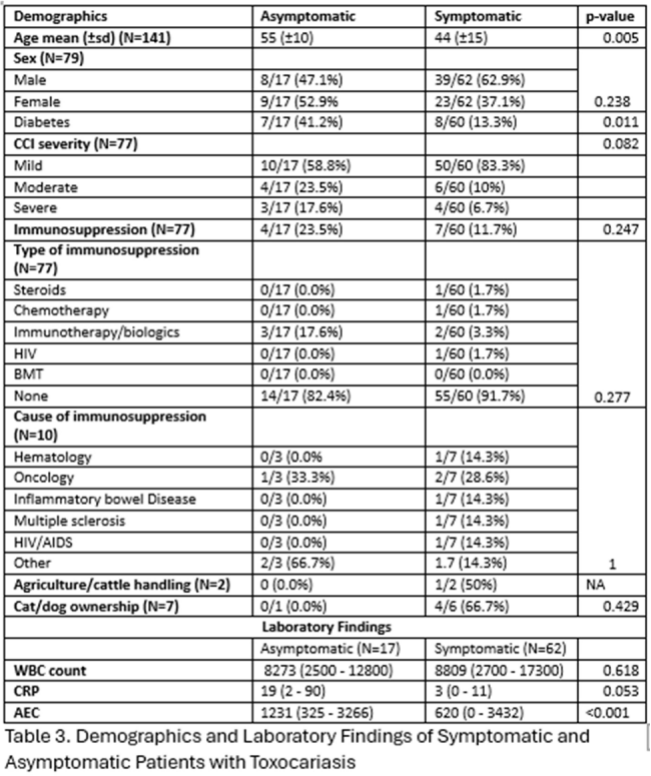

**Conclusion:**

According to our center's experience, most patient who presented were symptomatic, mainly complaining of neurologic symptoms, with those diagnosed with *Toxocara* myelitis mainly showing longitudinal enhancing lesions involving the thoracic levels. Further studies are crucial to ascertain the actual prevalence and clinical presentations of *Toxocara* in the region.

**Figure:**
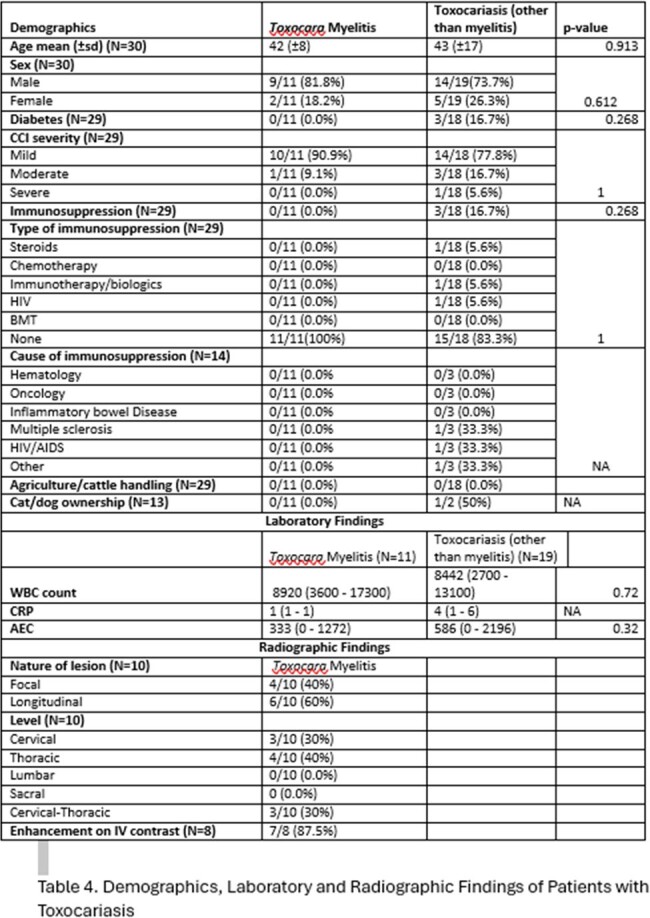

**Disclosures:**

**All Authors**: No reported disclosures

